# Selective Orexin Receptor Antagonists as Novel Augmentation Treatments for Major Depressive Disorder: Evidence for Safety and Efficacy From a Phase 2B Study of Seltorexant

**DOI:** 10.1093/ijnp/pyab078

**Published:** 2021-11-17

**Authors:** Manish Kumar Jha

**Affiliations:** Center for Depression Research and Clinical Care, Department of Psychiatry, UT Southwestern Medical Center, Dallas, Texas, USA

**Keywords:** Antidepressant, insomnia, major depressive disorder, Orexin, Seltorexant

## Abstract

There is a large unmet need for effective treatment of major depressive disorder (MDD), an often chronic/recurrent disorder that affects 1 in 5 adults during their lifetime in the United States. Clinicians and individuals with MDD often rely on augmentation approaches given the low rate of remission with the initial antidepressant treatment. Therefore, the report by Savitz and colleagues on the safety and efficacy of seltorexant is of great interest because it provides initial evidence for the antidepressant potential of drugs targeting orexin neurotransmission. Findings of this study suggest that seltorexant 20 mg is more effective than placebo, especially in individuals with moderate or insomnia symptoms at baseline. Given that insomnia is a common feature of depression, orexin 2 receptor antagonists may serve as important new treatment alternatives for people with MDD.

Major depressive disorder (MDD) affects 1 in 5 adults and is the second leading cause of disability in the United States (Vos et al., 2017; Hasin et al., 2018). Clinical outcomes with currently available treatments remain suboptimal because only 1 in 3 individuals with MDD attain remission with the initial antidepressant treatment (Trivedi et al., 2006b). Therefore, treatment regimens often incorporate augmentation of ongoing antidepressant with drugs such as second-generation antipsychotics or buspirone. This practice is supported by the findings of the Veterans Affairs Augmentation and Switching Treatments for Improving Depression Outcomes study, where acute-phase remission rates after nonresponse to at least 1 antidepressant treatment were higher with aripiprazole augmentation than with a switch to another antidepressant (bupropion) (Mohamed et al., 2017). Low remission rates with these augmentation strategies remains a major challenge (Trivedi et al., 2006a; Mohamed et al., 2017), thereby underscoring the need to develop new treatments. In this regard, the results of the phase 2b study of seltorexant (Savitz et al., 2021), a selective orexin 2 receptor antagonist (Bonaventure et al., 2015), are welcome because they provide early evidence for safety and efficacy of targeting orexin neurotransmission as a novel approach for treatment of MDD.

Orexins (or hypocretins) are peptide neurotransmitters simultaneously discovered by 2 independent groups (de Lecea et al., 1998; Sakurai et al., 1998). Sakurai and colleagues observed that central administration of these peptides stimulated feeding behavior in rats and named it orexin based on the Greek word *orexis*, which means appetite (Sakurai et al., 1998). de Lecea and colleagues used the term hypocretin to describe the same peptides based on their location in the hypothalamus and their similarity to the gut hormone secretin (de Lecea et al., 1998). The 2 orexin peptides, orexin-A and orexin-B (or hypocretin-1 and -2), are formed by proteolysis of the same precursor protein called prepro-orexin (de Lecea et al., 1998; Sakurai et al., 1998). Sakurai et al. (1998) also identified the 2 G protein coupled–receptors for which these orexin peptides are ligands and called them orexin 1 (OX1R; higher affinity for orexin-A) and orexin 2 (OX2R, similar affinity for both orexin-A and orexin-B) receptor, respectively. Emerging research over the past 2 decades has highlighted the therapeutic potential of drugs targeting orexin receptors, from the potential utility of agonists for narcolepsy to the Food and Drug Administration approval of suvorexant and lemborexant, both dual orexin receptor antagonists, for the treatment of insomnia (Sun et al., 2021). However, the efficacy of suvorexant and lemborexant in the treatment of depression remains poorly understood because phase 3 studies of both medications excluded individuals with depression (Herring et al., 2019; Kärppä et al., 2020). A 6-week, double-blind, phase 2 study of filorexant, a dual orexin receptor antagonist, was terminated early due to enrollment challenges and did not find any significant reduction in depression with filorexant compared with placebo (Connor et al., 2017). Therefore, the report by Savitz et al. (2021) is the first well-powered study to evaluate the antidepressant effect of an orexin receptor antagonist.

In their study, Savitz and colleagues recruited individuals with MDD who had <50% improvement in their depressive symptoms with at least 1 but no more than 3 antidepressants at an adequate dose and duration in their current major depressive episode. They also restricted eligibility to individuals who were on a stable dose of 1 of the prespecified selective serotonin reuptake inhibitors or serotonin and norepinephrine reuptake inhibitors for 4 weeks but not more than 12 months. The extensive set of eligibility criteria used by authors is similar to those in other antidepressant efficacy trials but raise questions about generalizability of their findings (Zimmerman et al., 2019). Authors provide detailed information in their Figure 1B (Savitz et al., 2021) that allows readers to understand the causes of screen failure. It is noteworthy that failure to meet inclusion criteria related to depression severity threshold was the most frequent cause of screen failure given that higher pretreatment depression severity has been linked to reduced placebo response (Trivedi et al., 2018).

Authors used an adaptive design because they wanted to identify a dose-response relationship. Therefore, the randomization scheme and dose of study medication were changed after a prespecified interim analysis. Although this approach allows for dose-response estimation with fewer number of individuals, none of the a priori identified standard models for dose-response relationship were statistically significant. The sigEmax model trended towards significance and thereby suggests an inverted U-shaped curve of dose-response relationship where a 20-mg seltorexant dose was more effective in reducing depressive symptoms than the 10-mg dose and had a trend toward greater improvement than the 40-mg dose. Whether the 20-mg seltorexant dose is superior to a 40-mg dose remains uncertain. For example, the number needed to treat for remission at week 6, that is, the number of additional individuals who need to be treated with the intervention compared with placebo to get 1 additional remission, was similar for the 20-mg and 40-mg doses of seltorexant (8 and 10, respectively). When stratified by the presence of moderate or severe insomnia at baseline, improvement in depressive symptoms was significantly greater with 20 mg seltorexant compared with placebo as well as with both 10-mg and 40-mg doses of seltorexant at week 3. At week 6, 20 mg seltorexant was more effective in improving depressive symptoms than placebo and the 10-mg seltorexant dose, but not the 40-mg seltorexant dose, in individuals with moderate or severe insomnia symptoms at baseline. In the absence of moderate or severe insomnia, improvement with the 20-mg dose of seltorexant was similar to placebo and other doses of seltorexant. Together, these findings seem to have informed the phase 3 studies of seltorexant, where a single dose of 20 mg is being evaluated in individuals with MDD who have moderate or severe insomnia at baseline and have responded inadequately to antidepressant therapy (Savitz et al., 2021).

Overall, seltorexant was well tolerated with similar rates of treatment-emergent adverse events as placebo. Somnolence was observed more frequently with the 20-mg seltorexant dose than placebo, which is consistent with previous studies of seltorexant (De Boer et al., 2018; van der Ark et al., 2018). Effects of chronic treatment with seltorexant on daytime sleepiness (and related functional impairments) need to be better characterized in the presence of comorbid disorders such as obstructive sleep apnea. A recent population-based study found that over 1 in 4 individuals with treatment-resistant depression who were treated with intranasal esketamine had a lifetime diagnosis of obstructive sleep apnea (Cepeda et al., 2021). Therefore, clinicians and researchers may need to systematically evaluate for these conditions, for example, by using tools such as the STOP-Bang Questionnaire for obstructive sleep apnea (Chung et al., 2016), when considering the use of seltorexant. Future studies are needed to understand the long-term consequences of treatment with seltorexant because certain side-effects such as weight gain may take weeks to months to gain clinical significance. Additionally, longer-term follow-up periods after treatment discontinuation will inform whether this class of medications is associated with any discontinuation symptoms (Jha 2019).

The development selective antagonists for OX1R [such as JNJ-61 393 215 (NCT04080752) (Salvadore et al., 2020) and AZD4041 (NCT04076540)] and OX2R (seltorexant) are of great interest to the scientific community. These medications offer the potential for treatment of various psychiatric disorders, ranging from MDD with insomnia symptoms (Savitz et al., 2021) or with anxious distress (NCT04080752) to cocaine (Hollander et al., 2012; James et al., 2021) or nicotine use disorder (Kenny 2011). Because bipolar disorder is often associated with sleep and circadian rhythm dysregulation (Bradley et al., 2017), future studies may evaluate the efficacy of OX2R antagonists for individuals with bipolar depression. This is especially important for bipolar depression, where only 4 medications have been approved by the Food and Drug Administration for acute treatment (Jha and Murrough 2019; Citrome 2020).

A recent preclinical study raises the potential of using OX2R antagonist for treatment of aggression (Flanigan et al., 2020). In their study, Flanigan and colleagues found that orexin neurons in the lateral hypothalamus activated glutamic acid decarboxylase 2–expressing neurons in the lateral habenula via OX2R in male mice, which, in turn, promoted male–male aggression and conditioned place preference for aggression-paired contexts (Flanigan et al., 2020). Furthermore, they found that systemic administration of an OX2R antagonist reduced male–male aggression and the conditioned place preference for aggression-paired context without affecting locomotion or anxiety-like behaviors (Flanigan et al., 2020). Given that aggression and its related construct of irritability are common transdiagnostic features (Jha et al., 2019; Eshel and Leibenluft 2020; Jha et al., 2021; Jha and Trivedi 2021), the availability of selective OX2R antagonists may help with experimental medicine studies that elucidate the neurocircuit mechanisms underpinning these features and help in the development of circuit-specific treatments.

In conclusion, the report by Savitz et al. (2021) is of great interest because it provides early evidence for safety and efficacy of seltorexant, a potential antidepressant drug with a novel mechanism of action. Given that insomnia is a common feature of MDD and that a majority of individuals with MDD do not respond adequately to the initial treatment, OX2R antagonists may serve as an important advance in the treatment of MDD.

## Statement of Interest

Dr Jha has received contract research grants from Acadia Pharmaceuticals and Janssen Research and Development; an educational grant to serve as Section Editor of the Psychiatry and Behavioral Health Learning Network; consultant fees from Eleusis Therapeutics US, Inc, Janssen Global Services, and Guidepoint Global; and honoraria from the North American Center for Continuing Medical Education and Global Medical Education.
